# Cancer Drugs Approved Based on Surrogate Endpoint: A Retrospective Observational Study in the United States and China

**DOI:** 10.1002/cam4.70864

**Published:** 2025-04-15

**Authors:** Ting Zhu, Jinjia Zhong, Yafang Huang

**Affiliations:** ^1^ School of General Practice and Continuing Education Capital Medical University Beijing China

**Keywords:** accelerated approval, cancer drug, regular approval, response rate, surrogate endpoint

## Abstract

**Background:**

Hundreds of cancer drugs are approved globally based on surrogate endpoint response rate (RR). However, the characteristics of RR‐based approvals remain unknown.

**Methods:**

In this retrospective study, all cancer drug‐indication pairs approved based on RR in the United States and China up to December 2023 were analyzed.

**Results:**

A total of 249 RR‐supported drug‐indication pairs were identified in the United States and 98 in China. In the United States, 98 of the 249 (39.4%) indications were granted regular approval (RA), whereas in China, only 21 of 98 (21.4%) approvals followed this regulatory pathway, with the remainder receiving accelerated approval (AA). The conversion rate from AA to RA was significantly lower in China compared to the United States (13.3% vs. 28.1%, *p* < 0.001). The proportion of AA withdrawals was significantly lower in China compared to the United States (1.0% vs. 10.4%, *p* < 0.001). Among all indications, the median RR in China was 60.9% (IQR, 35.8%–75.0%), which was significantly higher than the 45.0% (IQR, 29.0%–61.0%) in the United States (*p* < 0.001). In China, 18 of the 98 (18.4%) had an RR less than 30%. In contrast, in the United States, 26.9% of the 249 had an RR less than 30%.

**Conclusions:**

Compared to the United States, RR‐supported approvals in China are characterized by higher RR values and a stricter RA pathway. Regulatory authorities in both countries may need to consider both the quantity and quality during cancer drug development based on surrogate endpoints.

**Abbreviations**
AA: accelerated approval
CDE: Center for Drug Evaluation
DoR: duration of response
FDA: US Food and Drug Administration
ICI: immune checkpoint inhibitor
IQR: interquartile range
NMPA: National Medical Products Administration
NSCLC: non‐small cell lung cancer
OS: overall survival
PI: prescribing information
RA: regular approval
RCT: randomized controlled trial
RECIST: response evaluation criteria in solid tumors
RET: rearranged during transfection
RR: response rate
SMKI: small molecule kinase inhibitors
TKI: tyrosine kinase inhibitor

## 1 Introduction

Over the past decade, worldwide utilization of surrogate endpoints to preliminarily evaluate efficacy and expedite approval has become increasingly prevalent in the development of cancer drugs [1]. Particularly for the targeted drugs, those addressing novel mechanisms of action—such as pembrolizumab [2] targeting immune checkpoints and selpercatinib [3] targeting the novel cancer driver gene RET—surrogate endpoints in single‐arm trials have successfully supported their accelerated approvals (AAs) [4]. For patients with irreversible and life‐threatening cancers, the timely availability of these innovative therapies provides a crucial opportunity to extend life.

Nearly half of novel cancer drug indications have been approved by the US FDA based on surrogate endpoint response rate (RR, the percentage of patients whose tumors shrink beyond an arbitrary threshold), underscoring its significant role in drug clinical development [4, 5, 6]. However, the use of this surrogate measure RR for cancer drug approval remains poorly understood. As the world's second‐largest developer of innovative cancer drugs, China has made remarkable progress in recent years [7]. From 2016 to 2023, China has outpaced Europe and Japan in pivotal clinical trials for innovative drugs since 2020 and surpassed the United States in 2023 [8]. Currently, China accounts for nearly one‐third of the world's clinical trials [8]. Of the 418 pivotal trials for first‐in‐class (FIC) agents, more than half are focused on innovative oncology drugs [8, 9]. China officially became a member of the International Council for Harmonisation of Technical Requirements for Pharmaceuticals for Human Use (ICH) in 2017, suggesting that innovative drugs can be mutually recognized among member countries. Some original drugs developed in China, such as zanubrutinib [10, 11] and fruquintinib [12], have also been granted market approval by the US FDA. Moreover, China's regulation has undergone significant reforms to encourage innovative drug development, particularly for those irreversible and life‐threatening diseases, ensuring early access to treatment [8]. As China's pharmaceutical industry increasingly integrates with the global market, understanding the current evidence on surrogate endpoints is critical. Nevertheless, the investigation on surrogate endpoint supporting cancer drug approval in China is still lacking.

A previous study analyzing cancer drug approvals in the United States from 2006 to 2018 revealed that 86 indications were approved based on RR [6]. Furthermore, one‐third of these approvals had a supporting RR less than 30%, which appears less than promising [6]. However, since this study, no new related research has been conducted to date in the United States. Based on studies from other countries, for example, in Japan, 37.1% (111 out of 299) of cancer drug approvals between 2001 and 2020 were based on RR [13]. Another study of products authorized from 2011 to 2018 by the European Medicines Agency (EMA) found that most of the expedited approvals, 90% (46 out of 51) were based on surrogate endpoints [14]. With the rapid advancement of precision oncology and targeted therapies in recent years, the number of targeted cancer drugs, such as tazemetostat [15, 16] and pralsetinib [17, 18], approved based on RR has surged, highlighting an explosive growth trend.

Given that RR has supported numerous drug approvals in the United States, comparing the situation of RR‐supported drug approvals in China would be important for providing an international cross‐reference to facilitate policy‐making by regulatory authorities in both countries. This comparison also plays a meaningful role in fostering international regulatory harmonization and optimizing global cancer drug approvals. Furthermore, a surrogate endpoint does not mean an improvement in longevity [6], approval decisions mainly depend on the policies and views of regulatory experts. In this context, the study on the two countries is even more meaningful. This study aims to examine cancer drugs approved based on RR in the United States and China, the two leading countries in cancer drug development and the largest ICH member countries globally, to provide valuable global insights to help regulatory authorities worldwide in policy formulation.

## 2 Materials and Methods

### 2.1 Sample Identification

Cancer drugs and their indications approved in the United States were comprehensively reviewed and identified using the Drugs@FDA database (https://www.accessdata.fda.gov/scripts/cder/daf/) [19]. Cancer drugs approved in China were identified from the Chinese National Medical Products Administration (NMPA) and Center for Drug Evaluation (CDE) at (https://www.cde.org.cn/) [20]. Supporting information was manually retrieved from professional databases (PharmCube, DXY.cn, and the official websites of pharmaceutical manufacturers) in order to prevent missing or incomplete data.

For each drug, the full prescribing information (PI) and approval documents were retrieved. PI and approval documents were collected before December 31, 2023 for both NMPA‐ and FDA‐approved cancer drugs. Every approved drug and each of its subsequently approved new indications were considered as separate entries. We further reviewed the approval status of each indication through September 1, 2024 to identify which indications had been withdrawn or which accelerated approved indications had been converted to regular approval.

Based on the PI and approval documents, the identified indications were matched with the pivotal clinical trials supporting their approval. The pivotal clinical trial information was cross‐verified respectively using the ClinicalTrials.gov database of the US National Library of Medicine and the Chinese Clinical Trial Registry (ChiCTR) [21]. We included only cancer drug indications for which approval was based on RR endpoint, referred to as RR‐supported indications. The RR endpoint was defined based on previously published studies [5, 6]. In brief, tumor‐specific RR criteria were used to justify the drug approval. For solid tumors, Response Evaluation Criteria in Solid Tumors (RECIST) criteria were utilized. For hematologic malignancies, the RR was assessed based on PET scan results, clinical assessment, complete blood counts, serological testing, cytogenetic testing, and molecular response.

### 2.2 Data Extraction

We gathered indication information on conditional approval by the NMPA and AA by the FDA. Since both approvals share the same mechanism of expedited pathway that is based on surrogate endpoints, we used the term “accelerated approval” uniformly throughout this study to ensure clarity and consistency in reporting.

Data extraction was independently performed by two investigators (T.Z. and J.Z.), with any discrepancies resolved through discussion with a third investigator (Y.H.). The collected drug and indication data for both the NMPA and the FDA included: year of the approval, type of the first approval (regular approval, AA), study design used for the first approval (randomized controlled trial [RCT], non‐RCT), subsequent conversion to regular approval (yes, no), study design used for the subsequent conversion (RCT, non‐RCT), drug mechanism (small‐molecular kinase inhibitors [SMKIs], biological therapy, cytotoxic chemotherapy, others), cancer types (solid cancer, hematological malignancies), type of submission (initial, supplemental), type of administration (monotherapy, combination therapy), genome‐targeted therapies [22, 23] (yes, no), first‐line treatment (yes, no). For each approved cancer drug, detailed information on the drug target was also collected.

RR values were extracted from the pivotal trials that supported NMPA and the FDA approvals. Complete RR values were also extracted when available.

### 2.3 Statistical Analysis

Data were analyzed using descriptive statistics. To compare RR‐supported indications and their regulatory characteristics between the NMPA and FDA, the chi‐squared test was used. A Sankey diagram was created to visually display the distribution of cancer types, drug targets, and current approval status for both regulatory agencies. RR values were categorized into five groups based on their magnitude and presented in a tabular form [6]. Additionally, RRs were described using medians and interquartile ranges. Comparisons of RRs were conducted based on approval type. The Kruskal–Wallis test was used to compare RRs across three approval groups: AA only, AA followed by regular approval, and regular approval only. The Mann–Whitney test was used to compare RRs between two approval groups, including: first accelerated versus first regular approval, AA (to date) versus regular approval (to date or at any time). A *p* value of less than 0.05 was considered statistically significant. All statistical analyses were performed using SAS version 9.4 (SAS Institute, Cary, NC, USA), and the Sankey diagram was generated using R version 4.4.2.

## 3 Results

### 3.1 Comparison of RR‐Supported Indication and Their Regulatory Characteristics Between the NMPA and the FDA

A total of 559 FDA‐approved cancer drug indications were identified. Among them, 249 (44.5%) were RR‐supported, covering 137 cancer drugs. In comparison, 317 NMPA‐approved cancer drug indications were identified, with 98 (30.9%) being RR‐supported, covering 68 cancer drugs. Table 1 summarizes RR‐supported indications and their regulatory characteristics approved by the NMPA and FDA. The NMPA was likely to offer fewer regular approvals (21.4% vs. 39.4%; *p* = 0.0015), less conversion from AAs to regular approvals (13.3% vs. 28.1%; *p* = 0.0001). On the contrary, indications approved by NMPA were more likely to receive AA (78.6% vs. 60.6%; *p* = 0.0015), to be initial submissions (71.4% vs. 53.0%; *p* = 0.0017), and to involve genome‐targeted therapies [22, 23] (48.0% vs. 29.7%; *p* = 0.0013) compared to those approved by the FDA. No significant difference was observed between NMPA and the FDA approvals in the use of RCTs, either at the time of first approval (*p* = 0.6092) or at any point thereafter (*p* = 0.4342 for ever using RCTs). In total, the RR for supporting indication approvals by the NMPA was significantly higher (60.9% [IQR, 35.8%–75.0%] vs. 45.0% [IQR, 29.0%–61.0%]; *p* = 0.0005) than those approved by the FDA. In China and the United States, 88.8% and 86.7% of RR data, respectively, were generated from single‐arm trials rather than RCTs. The proportion of AA withdrawals was significantly lower in China compared to the United States (1.0% vs. 10.4%, *p* < 0.0001).

**TABLE 1:** Characteristics of RR‐supported approvals and indications granted by NMPA and the FDA.

	NMPA, *n* (%)	FDA, *n* (%)	*p* value
First approval status			
Regular	21 (21.4)	98 (39.4)	0.0015
Accelerated	77 (78.6)	151 (60.6)	
Conversion pathway			
Regular approval first	21 (21.4)	98 (39.4)	< 0.0001
Accelerated followed by regular	13 (13.3)	70 (28.1)	
Accelerated approval thus far	64 (65.3)	81 (32.5)	
Current approval status			
Regular approval	34 (34.7)	168 (67.5)	< 0.0001
Accelerated approval remaining	63 (64.3)	55 (22.1)	
Withdrawn	1 (1.0)	26 (10.4)	
Type of submission			
Initial	70 (71.4)	132 (53.0)	0.0017
Supplemental	28 (28.6)	117 (47.0)	
Cancer type			
Solid	56 (57.1)	127 (51.0)	0.3025
Hematologic	42 (42.9)	122 (49.0)	
Type of administration			
Monotherapy	83 (84.7)	203 (81.5)	0.4852
Combination therapy	15 (15.3)	46 (18.5)	
Genome‐targeted therapies			
Yes	47 (48.0)	74 (29.7)	0.0013
No	51 (52.0)	175 (70.3)	
First‐line therapy			
Yes	19 (19.4)	33 (13.3)	0.1495
No	79 (80.6)	216 (86.7)	
Use of RCT for first approval			
Yes	11 (11.2)	33 (13.3)	0.6092
No	87 (88.8)	216 (86.7)	
Use of RCT ever			
Yes	22 (22.4)	66 (26.5)	0.4342
No	76 (77.6)	183 (73.5)	
Drug mechanism class			
SMKI	46 (46.9)	96 (38.6)	0.0800
Biologic therapy	41 (41.8)	95 (38.2)	
Cytotoxic chemotherapy	5 (5.1)	31 (12.4)	
Others	6 (6.1)	27 (10.8)	
Response rate end point leading to approval, median (IQR), %	60.9 (35.8–75.0)	45.0 (29.0–61.0)	0.0005
Reported median duration of response, median (IQR), mo	14.6 (9.4–18.0)	11.1 (8.2–16.7)	0.1238
Complete response rate only, median (IQR), %	7.3 (1.9–25.6)	11.0 (4.0–24.1)	0.1937
Sample size of initial drug registration trial, median (IQR), persons	95.5 (70.0–136.0)	97.0 (61.0–137.0)	0.5695

Abbreviations: FDA, US Food and Drug Administration; IQR, interquartile range; NMPA, National Medical Products Administration; RCT, randomized clinical trial; SMKI, small‐molecular kinase inhibitor.

Figure S1 showed the distribution of cancer type, drug target, and the approval status for RR‐supported indications approved by the NMPA and the FDA. No statistically significant differences were observed between the NMPA and FDA in terms of cancer type distribution (*p* = 0.4260) or drug target distribution (*p* = 0.5130).

### 3.2 Ranging RR Values Based on Their Magnitude

The median RR for supporting indication approvals by the NMPA was 60.9% (interquartile range [IQR], 35.8%–75.0%). Among the 98 NMPA approved indications, 5 (5.1%) had an RR less than 20%, 18 (18.4%) had an RR less than 30%, 29 (29.6%) had an RR less than 40%, and 36 (36.7%) had an RR range from 60% to 79% (Table 2). For complete response, the median complete RR across 70 available indications was 7.3% (IQR, 1.9%–25.6%), with 54 of 70 (77.1%) having a complete RR less than 30% (Table S1).

**TABLE 2:** Ranging RRs in quintiles based on their magnitude.

	Drug response rate in quintiles, *N* (%)					
	80–100	60–79	40–59	20–39	0–19	Total
NMPA						
Regular approval	6 (28.6)	8 (38.1)	4 (19.0)	3 (14.3)	0	21
Accelerated then OS approval	0	0	0	1 (50.0)	1 (50.0)	2
Accelerated then PFS approval	0	6 (66.7)	1 (11.1)	2 (22.2)	0	9
Accelerated then RR approval	0	1 (50.0)	1 (50.0)	0	0	2
Accelerated only	9 (14.1)	21 (32.8)	12 (18.8)	18 (28.1)	4 (6.3)	64
Total	15 (15.3)	36 (36.7)	18 (18.4)	24 (24.5)	5 (5.1)	98
FDA						
Regular approval	14 (14.3)	21 (21.4)	29 (29.6)	24 (24.5)	10 (10.2)	98
Accelerated then OS approval	0	1 (7.1)	5 (35.7)	4 (28.6)	4 (28.6)	14
Accelerated then PFS approval	1 (4.5)	5 (22.7)	9 (40.9)	5 (22.7)	2 (9.1)	22
Accelerated then RR approval	4 (11.8)	8 (23.5)	7 (20.6)	11 (32.4)	4 (11.8)	34
Accelerated only	3 (3.7)	15 (18.5)	27 (33.3)	27 (33.3)	9 (11.1)	81
Total	22 (8.8)	50 (20.1)	77 (30.9)	71 (28.5)	29 (11.6)	249

Abbreviations: FDA, US Food and Drug Administration; NMPA, National Medical Products Administration; OS, overall survival; PFS, progression‐free survival; RR, response rate.

In comparison, the median RR for supporting indication approvals by the FDA was 45.0% (IQR, 29.0%–61.0%). Among the 249 RR‐supported indications approved by the FDA, 29 (11.6%) had an RR less than 20%, 67 (26.9%) had an RR less than 30%, 100 (40.1%) had an RR less than 40%, and 77 (30.9%) had an RR range from 40% to 59% (Table 2). Regarding complete response, the median complete RR across 197 available indications was 11.0% (IQR, 4.0%–24.1%), with 157 of 197 (79.7%) having a complete RR less than 30% (Table S1).

### 3.3 Comparison of RRs Across Different Approval Statuses

A significant difference in RR was observed between NMPA‐approved indications granted AA and those granted regular approval (median, 57.8%; IQR, 34.0%–73.8% vs. median, 67.6%; IQR, 51.5%–83.5%; *p* = 0.0455). The RR for NMPA‐approved indications granted AA pending confirmation was not statistically significantly lower than for indications converted to regular approval or those initially granted regular approval (median, 53.9%; IQR, 33.4%–76.3% vs. median, 68.0%; IQR, 37.0%–70.0% vs. median, 67.6%; IQR, 51.4%–83.5%; *p* = 0.1340) (Figures S2–S4). No significant difference in the RR was observed between NMPA‐approved indications with pending confirmation of AA and those that had obtained regular approval, either initially or following conversion (median, 53.9%; IQR, 33.4%–76.3% vs. median, 67.8%; IQR, 50.8%–74.1%; *p* = 0.1606) (Table 3). A comparison of complete RRs across NMPA approval statuses is provided in Table S2.

**TABLE 3:** Median RRs in each type of approval status in NMPA and FDA.

	Median (IQR) [range]	N	*p* value
RR by first approval type (NMPA)			
Drugs granted RA first	67.6 (51.4–83.5) [22.9–93.8]	21	0.0455
Drugs granted AA first	57.8 (34.0–73.8) [13.3–100.0]	77	
RR by conversion status of AAs (NMPA)			
Drugs granted RA first	67.6 (51.4–83.5) [22.0–93.8]	21	0.1340
Drugs granted accelerated followed by RA	68.0 (37.0–70.0) [13.3–74.1]	13	
Drugs granted only AA thus far	53.9 (33.4–76.3) [16.7–100.0]	64	
RR by current approval status (NMPA)			
Drugs already granted RA thus far	67.8 (50.8–74.1) [13.3–93.8]	34	0.1606
Drugs granted only AA thus far	53.9 (33.4–76.3) [16.7–100.0]	64	
RR by first approval type (FDA)			
Drugs granted RA first	49.5 (31.0–71.5) [9.0–98.0]	98	0.0200
Drugs granted AA first	43.0 (28.0–59.0) [8.5–86.0]	151	
RR by conversion status of AAs (FDA)			
Drugs granted RA first	49.5 (31.0–71.5) [9.0–98.0]	98	0.0665
Drugs granted accelerated followed by RA	43.8 (29.0–60.0) [8.5–86.0]	70	
Drugs granted only AA thus far	42.2 (28.0–58.0) [11.76–85.1]	81	
RR by current approval status (FDA)			
Drugs already granted RA thus far	46.4 (29.2–66.0) [8.5–98.0]	168	0.1781
Drugs granted only AA thus far	42.2 (28.0–58.0) [11.76–85.1]	81	

Abbreviations: AA, accelerated approval; FDA, US Food and Drug Administration; IQR, interquartile range; NMPA, National Medical Products Administration; RA, regular approval; RR, response rate.

The RR for supporting FDA approvals of the indications granted AA was lower than for those granted regular approval (median, 43.0%; IQR, 28.0%–59.0% vs. median, 49.5%; IQR, 31.0%–71.5%; *p* = 0.0200). The RR for supporting FDA approvals of the indications granted AA pending confirmation was not significantly lower than for indications converted to regular approval or those initially granted regular approval (median, 42.2%; IQR, 28.0%–58.0% vs. median, 43.8%; IQR, 29.0%–60.0% vs. median, 49.5%; IQR, 31.0%–71.5%; *p* = 0.0665) (Figures S5–S7). No significant difference in the RR was observed between FDA‐approved indications with pending confirmation of AA and those that had obtained regular approval, either initially or following conversion (median, 42.2%; IQR, 28.0%–58.0% vs. median, 46.4%; IQR, 29.2%–66.0%; *p* = 0.1781) (Table 3). A comparison of complete RRs across FDA approval statuses is provided in Table S2.

Of the 249 FDA‐approved indications, 118 were approved after 2018. The RR was significantly higher in indications approved after 2018 compared with indications approved before 2018 (median, 46.8%; IQR, 35.0%–69.0% vs. median, 42.2%; IQR, 23.5%–59.0%; *p* = 0.0025).

## 4 Discussion

This study provides a retrospective review of nearly 350 RR‐supported approvals of cancer drug indications, including 249 indications for 137 drugs in the United States and 98 indications for 68 drugs in China. In both countries, these RR‐supported approvals covered a broad spectrum of oncology therapies, including indications for solid tumors and hematologic cancers, with drugs spanning SMKIs, biologics, and cytotoxic drugs. This indicates that surrogate endpoint RR has been extensively utilized to support the approval of cancer drugs in both China and the United States. For both countries, more than 80% of RR data were generated from single‐arm trials rather than RCTs. By comparing the data from both countries, we have identified several key findings that may reflect the differing attitudes of each country toward using RR as evidence to support the approval of cancer drugs.

First, we found that, compared to the United States, China appears to take a more stringent regulation toward using RR data to support drug approvals. This is primarily reflected in three key aspects: (1) There was a significant difference in the proportion of cancer drug indications approved based on RR between the United States and China (44.5% vs. 30.9%). (2) The number of cancer drug indications approved based on RR in China was only about 40% of that in the United States, although China has one of the largest cancer patient populations in the world. Since RR is a surrogate endpoint that only indicates anticancer activity such as tumor shrinkage, without guaranteeing improved longevity, this suggests a more cautious attitude toward such approvals in China. (3) Even when RR is used as supporting evidence, the China NMPA was less likely to grant regular approvals and had a lower conversion rate from AAs to regular approvals compared to the US FDA. In China, only 21 of 98 (21.4%) indications received regular approval, while in the United States, 98 of 249 (39.4%) indications were granted regular approval. China's conversion rate from AA to RA was significantly lower at 13.3%, compared to 28.1% in the United States. Moreover, the indications granted RA in China had a median RR of 67.6%, compared to 49.5% in the United States. These data implied that, compared to the United States, it is more challenging to obtain regular approvals (RA) in China using RR. In terms of quantity, the number of cancer drug indications approved based on RR and granted RA in the United States was nearly five times that in China. In Japan, it was reported that 37.1% of the total cancer drug approvals were on the basis of RR evidence [13]. In Europe, it was found that 90% of the expedited cancer drug approvals were on the basis of surrogate endpoints [14]. However, the magnitude of RR was not reported in these previous studies [13, 14].

Second, we found that compared to the United States, China regulation tends to require higher RR magnitude to support approval. This is evident in the following key aspects: (1) This study identified a significant difference in the RR value supporting the approval of cancer drug indication between China and the United States. Drugs approved based on RR in China had a significantly higher median RR of 60.9% (IQR, 35.8%–75.0%), compared to 45.0% (IQR, 29.0%–61.0%) in the United States. (2) Regarding the range of RR magnitude, in China, 18 of 98 (18.4%) indications had an RR below 30%, 29.6% had an RR below 40%, and most approved drugs had an RR range of 60% to 79%. In contrast, in the United States, 26.9% of 249 indications had an RR below 30%, 40.1% had an RR below 40%, and most approved drugs had an RR ranging from 40% to 59%. These differences suggest that cancer drugs approved based on RR evidence in China generally demonstrate higher RR magnitudes compared to those in the United States. (3) The median RR supporting regular approvals in China had reached an impressive 68%, significantly exceeding the 58% median RR supporting AAs.

It is important to indicate that RR, as a surrogate endpoint, reflects anticancer activity but does not mean that survival time was prolonged. The use of a 30% RR is an arbitrary cutoff and not a standardized threshold [5, 6]. As a result, approving drugs using RR often involves considerable subjectivity, largely depending on each country's regulatory policies and the perspectives of its expert reviewer. China has significantly improved its regulatory efficiency in the past decades, with more expedited pathways available, including the introduction of the expedited approval program in 2017—has led to a considerable increase in the number of newly approved cancer drugs [24]. These reforms reflect China's efforts to regulate drug approval pathways to address the timely clinical need for innovative therapies. Certainly, China's NMPA prefers to emphasize on ensuring clinical benefit in pre‐approval evidence requirements. The reliance on surrogate endpoints like RR in expedited approvals suggests the need to confirm clinical benefits during post‐marketing. Higher RR may predict more possibility in obtaining result of clinical benefit in post‐marketing studies. While post‐marketing studies are a common global practice, China's regulatory framework may place more emphasis on them, given the need for long‐term evidence in a rapidly evolving healthcare landscape. Studying RR‐supported approval in a single country may not reveal the regulatory outcomes of different authorities and attitudes. In contrast, comparing two major pharmaceutical nations might shed light on these differences. This comparison is not intended to determine whether the policies or perspectives of one country are better than those of another but rather to identify areas where mutual learning can be achieved by drawing on each other's experiences. A report indicated that the number of drug clinical trials in China surpassed those in Europe and Japan by 2020 and exceeded those in the United States by 2023 [8]. Over 200 innovative oncology drugs are currently undergoing clinical trials in China [9]. However, the number of cancer drug indications approved based on RR in China remains relatively limited.

Furthermore, the proportion of AA withdrawals in China was only 1.0%, while it was 10.4% in the United States. In recent years, attention and concerns regarding cancer drug approval based on surrogate endpoints have increased [25]. A previous report showed that between 2021 and 2022, up to 11 approved oncology indications were withdrawn in the United States [22]. The United States has strengthened its regulations in this area [25]. Additionally, our study revealed an improvement in the median RR supporting approvals in the United States in recent years. Before 2018, the median RR was 42.2%; however, it rose significantly to 46.8% after 2018. The proportion of indications approved with an RR below 30% decreased to 26%, compared to the previously reported 33% [6]. Last but not least, this study found a significant difference between China and the United States was that the number of RR‐supported indications with genomic biomarkers [22, 23] for personalized treatment is significantly higher in China. In China, these indications account for nearly half of the total, whereas in the United States, they account for less than 30%. It is known that genomic biomarker‐directed indications, such as crizotinib treating ALK positive non‐small cell lung cancer [26], have strength in tailoring treatments for individual patients by testing their gene alterations.

The validated surrogate endpoint which could better align with OS is very important. While RR serves as an important surrogate endpoint, future research should focus on improving RR's ability to predict clinically benefits. Moreover, assessment of RR in trials could better be conducted by an independent review committee (IRC) since the pivotal trial could be single‐arm and open‐label [27]. These aspects should be fully considered when designing the trials. Last but not least, given the large number of cancer drugs approved based on RR in various countries, regulating post‐marketing studies is crucial. It is particularly important to generate OS evidence through RCTs in these studies. For the regulatory agency in each country, it is recommended to implement specific measures to mitigate the risk of RR‐supported approvals. For example, it could be demonstrated that RR is a validated surrogate endpoint for the drug's mechanism of action and the cancer type. Additionally, an RCT with OS could be initiated before the drug is approved, with sponsors required to provide a timeline for trial completion. Finally, regulatory approval could require the involvement of an IRC or a blinded IRC in RR assessment to minimize result bias in the open‐labeled trial.

## 5 Conclusions and Policy Implication

In summary, for a country like China, with a large cancer patient population and significant unmet need for novel drugs, optimizing the regulatory strategy for RR‐supported accelerate approvals may help to facilitate timely access to innovative therapies, thereby promoting approval of the potential cancer therapies to better address clinical needs. Together, the United States and China, as the world's two largest innovators in cancer drug development and the largest ICH member countries [8, 9], have collectively achieved nearly 350 RR‐supported indication approvals. This number is expected to continue its upward trend [28]. Since surrogate endpoint does not mean an extension of survival and approval is influenced by regulatory authorities and policy experts, these authorities and experts in both countries may require considering a balance between quantity and quality when relying on surrogate endpoint as evidence. This study provides global insights from different countries on RR endpoint to support cancer drug approvals, assisting regulatory authorities worldwide in policy formulation in appraisal of RR evidence for cancer drug development.

## 6 Limitations

First, this study did not include data from Europe and Japan, and we did not describe or summarize cancer drugs approved based on RR in these regions. Second, RR only represents anticancer activity and does not fully reflect clinical benefits in terms of overall survival outcome, even though hundreds of RRs have been used in new drug approvals. The disconnection between RR and survival benefits raises concerns about the actual therapeutic value of drugs approved based on this surrogate endpoint. Further research is needed to evaluate the long‐term clinical impact of RR‐based approvals and validate the RR that could predict clinical benefit for patients with cancer. Third, as the approval status of newly marketed cancer drugs and their corresponding indications continues to evolve over time, the data presented in this study only reflect the situation up to the specified cutoff date. Future updates are necessary to continuously monitor the approval status of drugs based on RR and track the transition of AAs to regular approvals.

## Author Contributions

Data curation; investigation; formal analysis; visualization; writing – original draft; writing – review and editing: Ting Zhu. Software; data curation; investigation; validation; formal analysis; writing – review and editing: Jinjia Zhong. Conceptualization; methodology; software; data curation; investigation; validation; formal analysis; supervision; funding acquisition; visualization; project administration; resources; writing – original draft; writing – review and editing: Yafang Huang.

## Ethics Statement

This study was based on publicly available information and involved no patient records. Ethical approval was not required.

## Conflicts of Interest

The authors declare no conflicts of interest.

## Supporting information


Data S1.

## Data Availability

The data that support the findings of our study are available from the corresponding author upon reasonable request.
