# Supplementary material for: Cancer Drugs Approved Based on Surrogate Endpoint: A Retrospective Observational Study in the United States and China
Source: Cancer Med. 2025 Apr 15;14(8):e70864. doi: 10.1002/cam4.70864 (PMC11997453; doi:10.1002/cam4.70864)
Supplement: Supplementary file 1 — Data S1. [file CAM4-14-e70864-s001.docx]

**Supplementary Online Content**

**eTable 1. Ranging complete response rate in quintiles based on their magnitude**

**eTable 2. Median of complete response rates in each type of approval status**

**eFigure 1. The regulatory status of RR-supported approvals by NMPA and the FDA**

**eFigure 2. Median and range of RR supporting regular approval at first-time of indications by NMPA**

**eFigure 3. Median and range of RR supporting accelerated approval followed by regular approval of indications by NMPA**

**eFigure 4. Median and range of RR supporting accelerated approval pending verification by NMPA**

**eFigure 5. Median and range of RRs supporting regular approvals at first-time by the FDA**

**eFigure 6. Median and range of RRs supporting regular approvals converted from accelerated approvals by the FDA**

**eFigure 7. Median and range of RRs supporting accelerated approval pending verification by the FDA**

**eTable 1. Ranging complete response rate in quintiles based on their magnitude**

|  | **Drug complete response rate in quintiles, No. (%)** | | | | | |
| --- | --- | --- | --- | --- | --- | --- |
|  | **80-100** | **60-79** | **40-59** | **20-39** | **0-19** | **Totals** |
| **NMPA** |  |  |  |  |  |  |
| Regular approval | 0 | 2 (14.3) | 1 (7.1) | 3 (21.4) | 8 (57.1) | 14 |
| Accelerated then OS approval | 0 | 0 | 0 | 1 (50.0) | 1 (50.0) | 2 |
| Accelerated then PFS approval | 0 | 0 | 0 | 0 | 5 (100.0) | 5 |
| Accelerated then RR approval | 0 | 0 | 0 | 1 (50.0) | 1 (50.0) | 2 |
| Accelerated only | 0 | 2 (4.3) | 4 (8.5) | 8 (17.0) | 33 (70.2) | 47 |
| Total | 0 | 4 (5.7) | 5 (7.1) | 13 (18.6) | 48 (68.6) | 70 |
| **FDA** |  |  |  |  |  |  |
| Regular approval | 3 (4.1) | 4 (5.4) | 7 (9.5) | 17 (23.0) | 43 (58.1) | 74 |
| Accelerated then OS approval | 0 | 0 | 1 (7.7) | 1 (7.7) | 11 (84.6) | 13 |
| Accelerated then PFS approval | 0 | 1 (6.7) | 0 | 3 (20.0) | 11 (73.3) | 15 |
| Accelerated then RR approval | 2 (6.9) | 3 (10.3) | 3 (10.3) | 3 (10.3) | 18 (62.1) | 29 |
| Accelerated only | 0 | 1 (1.5) | 4 (6.1) | 8 (12.1) | 53 (80.3) | 66 |
| Total | 5 (2.5) | 9 (4.6) | 15 (7.6) | 32 (16.2) | 136 (69.0) | 197 |

Abbreviations: FDA, US Food and Drug Administration; NMPA, National Medical Products Administration; OS, overall survival; PFS, progression-free survival; RR, response rate.

**eTable 2. Median of complete response rates in each type of approval status**

|  | **Median (IQR) [Range]** | **No.** | ***P* Value** |
| --- | --- | --- | --- |
| **RR by first approval type (NMPA)** |  |  | 0.1838 |
| Drugs granted RA first | 17.3(4.5-35.8) [0-75.2] | 14 |  |
| Drugs granted AA first | 6.7(1.7-23.3) [0-77.9] | 56 |  |
| **RR by conversion status of AAs (NMPA)** |  |  | 0.4091 |
| Drugs granted RA first | 17.3(4.5-35.8) [0-75.2] | 14 |  |
| Drugs granted accelerated followed by RA | 5.7(2.8-13.8) [1.2-37.0] | 9 |  |
| Drugs granted only AA thus far | 7.0(1.5-24.7) [0-77.9] | 47 |  |
| **RR by current approval status (NMPA)** |  |  | 0.3547 |
| Drugs already granted RA thus far | 11.7(2.8-34.9) [0-75.2] | 23 |  |
| Drugs granted only AA thus far | 7.0(1.5-24.7) [0-77.9] | 47 |  |
| **RR by first approval type (FDA)** |  |  | 0.0006 |
| Drugs granted RA first | 15.3(8.0-35.0) [0-96.1] | 74 |  |
| Drugs granted AA first | 9.0(2.8-18.0) [0-87.0] | 123 |  |
| **RR by conversion status of AAs (FDA)** |  |  | 0.0012 |
| Drugs granted RA first | 15.3(8.0-35.0) [0-96.1] | 74 |  |
| Drugs granted accelerated followed by RA | 10.6(3.3-32.0) [0-87.0] | 57 |  |
| Drugs granted only AA thus far | 8.0(2.7-15.4) [0-60.0] | 66 |  |
| **RR by current approval status (FDA)** |  |  | 0.0028 |
| Drugs already granted RA thus far | 12.0(6.0-32.4) [0-96.1] | 131 |  |
| Drugs granted only AA thus far | 8.0(2.7-15.4) [0-60.0] | 66 |  |

Abbreviations: AA, accelerated approval; FDA, US Food and Drug Administration; IQR, interquartile range; NMPA, National Medical Products Administration; RA, regular approval; RR, response rate.

**eFigure 1. The regulatory status of RR-supported approvals by NMPA and the FDA**

Column 1 (left): Cancers. Column 2: Drugs approved by China NMPA and the US FDA. Column 3: Drug category based on their mechanisms, such as TKI and BTK. Column 4: The regulatory status. "AA follow by RA": have transitioned from accelerated approval to regular approval; "AA thus far": remain under accelerated approval without transition; "Withdrawn": indication approvals withdrawn. “RA first”: obtained regular approval at first-time.

Abbreviations: AKT, AKT serine/threonine kinase; BTK, Bruton’s tyrosine kinase; CAR-T, chimeric antigen receptor T-cell; ERK, extracellular signal-regulated kinase; ESCC, esophageal squamous cell carcinoma; GEJ, gastroesophageal junction; GIST, gastrointestinal stromal tumor; MAPK, mitogen-activated protein kinase; mTOR, mechanistic target of rapamycin; PI3K, phosphoinositide 3-Kinase; RA, regular approval; SMKI, small molecule kinase inhibitors; TKI, Tyrosine kinase inhibitor.

**
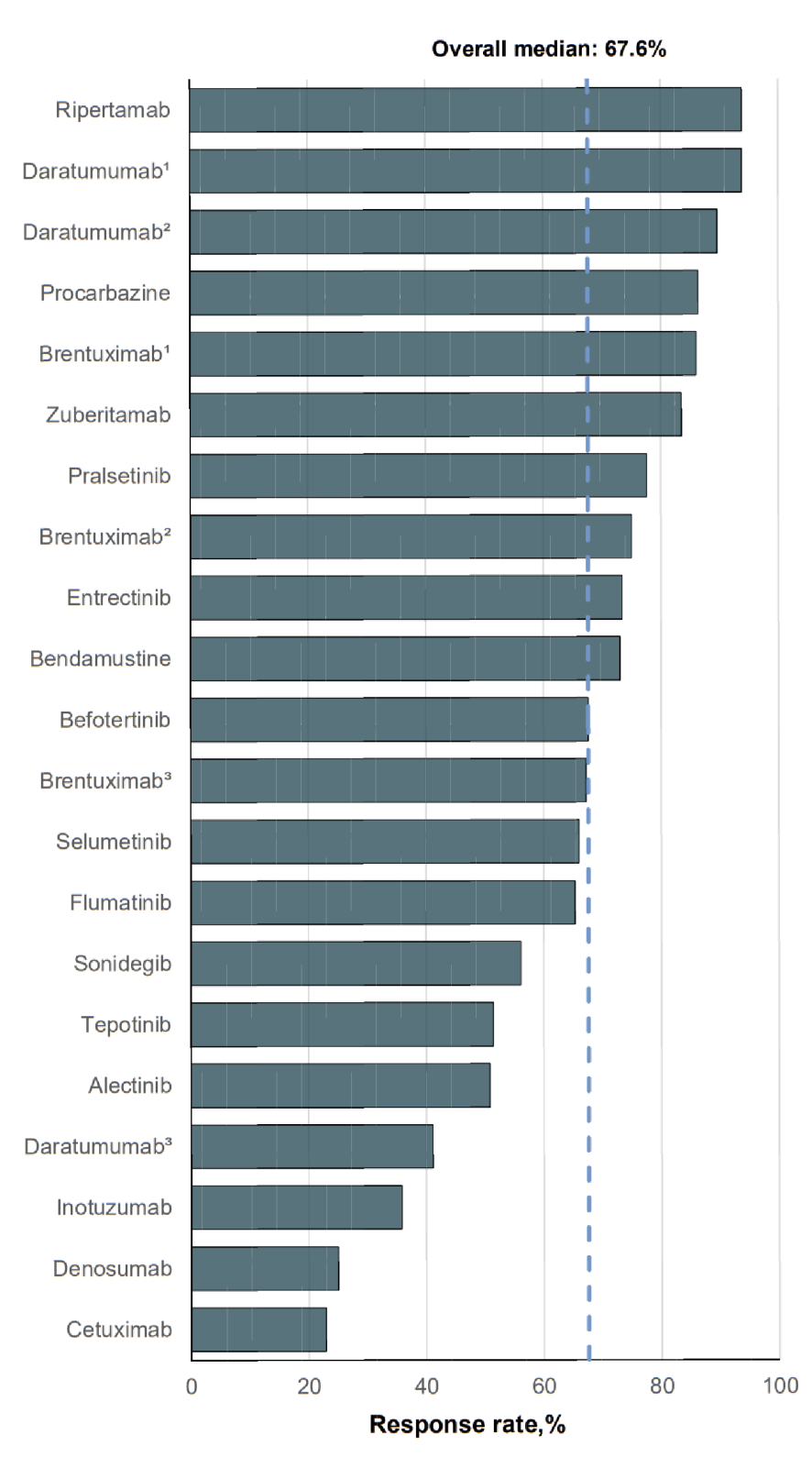
**

**eFigure 2. Median and range of RR supporting regular approval at first-time of indications by NMPA**

Daratumumab was granted three indications: Daratumumab¹ for multiple myeloma (MM) with prior therapy, Daratumumab² for newly diagnosed multiple myeloma, and Daratumumab³ for relapsed and refractory multiple myeloma in adults. Brentuximab was granted three indications: Brentuximab^1,2,3^ are respectively for the treatment of CD30-positive relapsed or refractory systemic anaplastic large cell lymphoma (sALCL), classical Hodgkin lymphoma (cHL), and primary cutaneous anaplastic large cell lymphoma (pcALCL) or mycosis fungoides (MF).

**eFigure 3. Median and range of RR supporting accelerated approval followed by regular approval of indications by NMPA**

**eFigure 4. Median and range of RR supporting accelerated approval pending verification by NMPA**


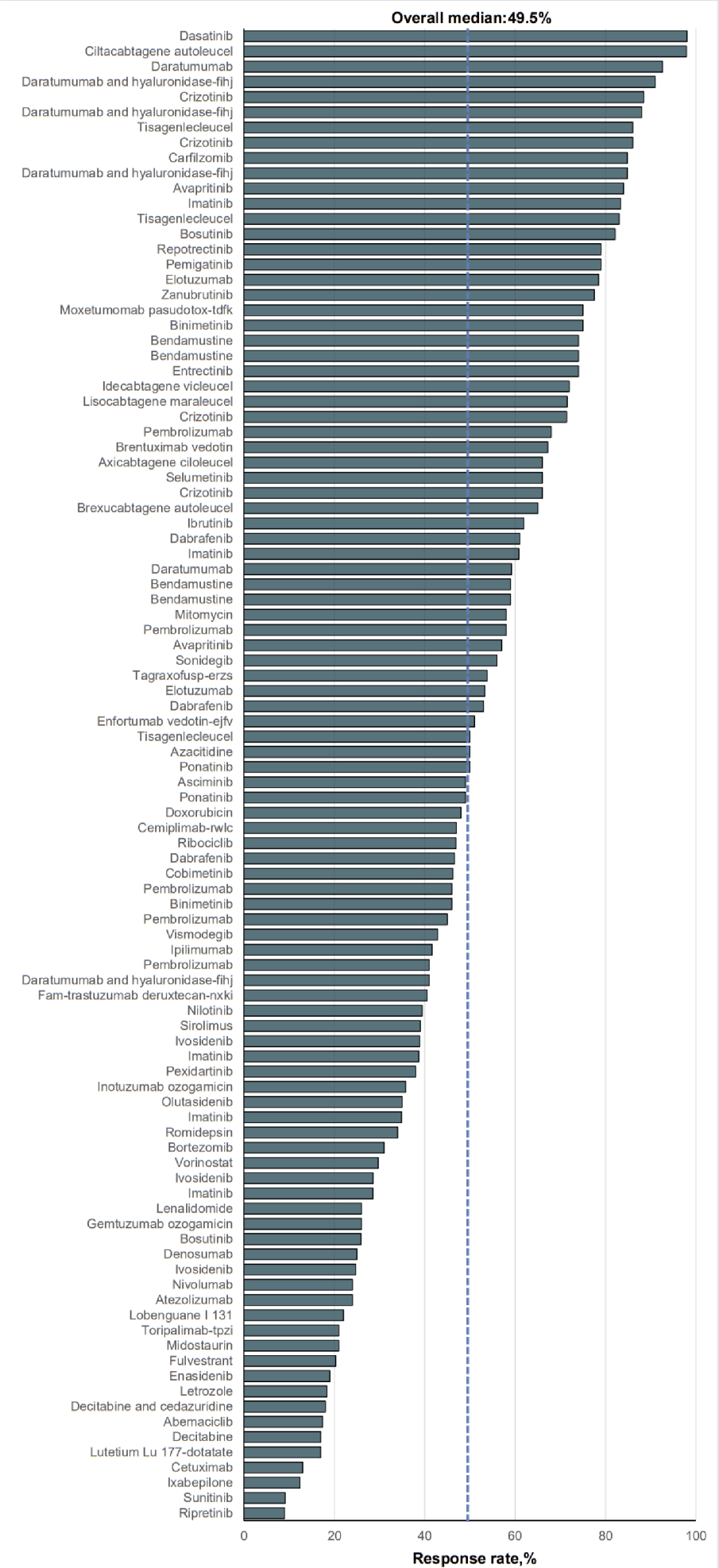


**eFigure 5. Median and range of RRs supporting regular approvals at first-time by the FDA**

**
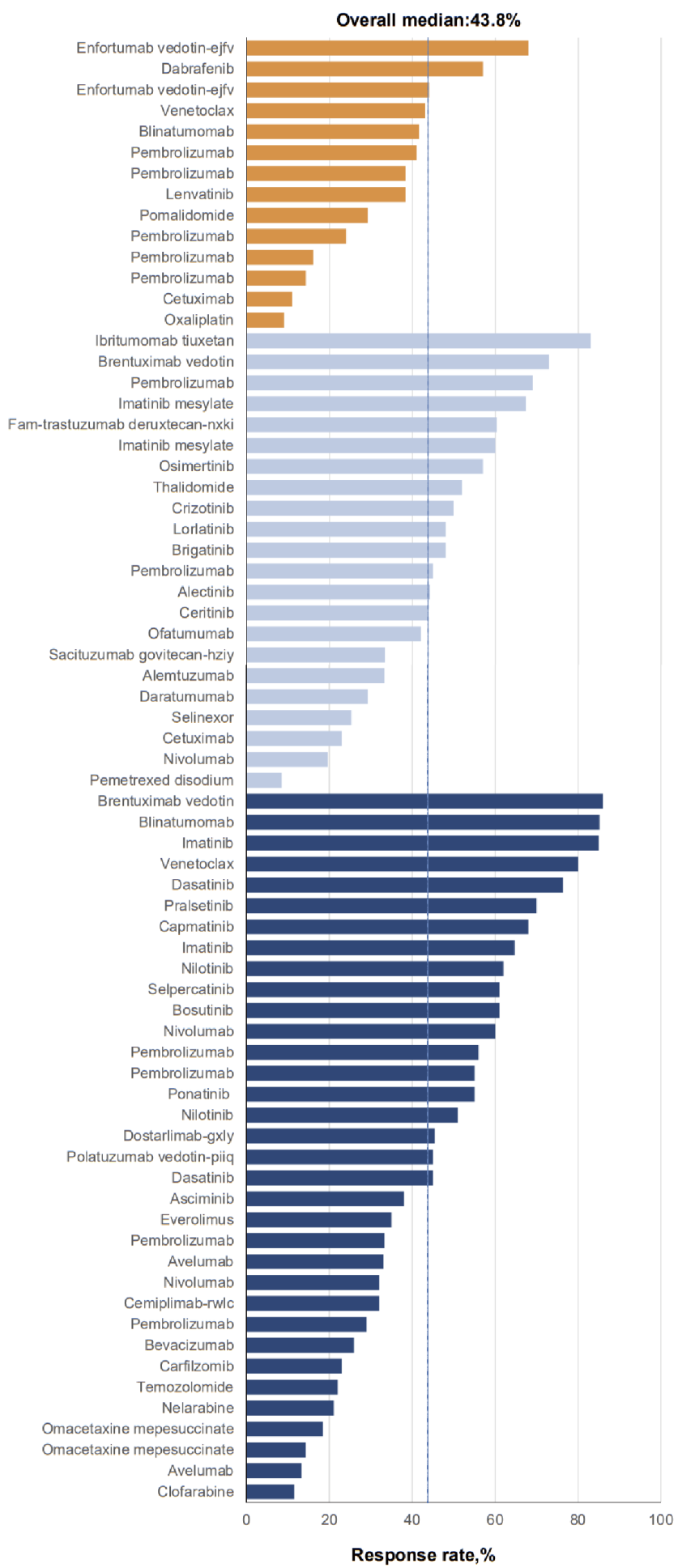
**

**eFigure 6. Median and range of RRs supporting regular approvals converted from accelerated approvals by the FDA**

**eFigure 7. Median and range of RRs supporting accelerated approval pending verification by the FDA**
